# Acute myeloid leukaemia: a paradigm for the clonal evolution of cancer?

**DOI:** 10.1242/dmm.015974

**Published:** 2014-08

**Authors:** Carolyn S. Grove, George S. Vassiliou

**Affiliations:** Haematological Cancer Genetics, The Wellcome Trust Sanger Institute, Wellcome Trust Genome Campus, Hinxton, Cambridge CB10 1SA, UK.

**Keywords:** Acute myeloid leukaemia, Cancer, Clonal evolution, *In vivo* models of leukaemia, Mutation

## Abstract

Acute myeloid leukaemia (AML) is an uncontrolled clonal proliferation of abnormal myeloid progenitor cells in the bone marrow and blood. Advances in cancer genomics have revealed the spectrum of somatic mutations that give rise to human AML and drawn our attention to its molecular evolution and clonal architecture. It is now evident that most AML genomes harbour small numbers of mutations, which are acquired in a stepwise manner. This characteristic, combined with our ability to identify mutations in individual leukaemic cells and our detailed understanding of normal human and murine haematopoiesis, makes AML an excellent model for understanding the principles of cancer evolution. Furthermore, a better understanding of how AML evolves can help us devise strategies to improve the therapy and prognosis of AML patients. Here, we draw from recent advances in genomics, clinical studies and experimental models to describe the current knowledge of the clonal evolution of AML and its implications for the biology and treatment of leukaemias and other cancers.

## Introduction

Acute myeloid leukaemia (AML) is an aggressive malignancy characterised by a block in myeloid differentiation [the process normally responsible for the generation of mature blood cells from haemopoietic stem cells (HSCs)] and uncontrolled proliferation of abnormal myeloid progenitors that accumulate in the bone marrow and blood. Some cases develop from other haematopoietic disorders or follow genotoxic therapy for unrelated malignancies, but most arise *de novo* (Østgård et al., 2010). Several genetic markers have been identified to stratify patients into prognostic groups, which are used to guide treatment decisions. Although chemotherapy results in high rates of remission, the majority of patients relapse and the overall 5 year survival is only 40–45% in young patients and less than 10% in the elderly (Craddock et al., 2005; Schlenk and Döhner, 2013). For a number of reasons, our understanding of the evolution and pathogenesis of AML has benefited particularly from recent advances in genomics, haemopoietic stem cell biology and studies using *in vivo* models. In this Review, we examine how these developments both illuminate the events and processes underlying the evolution of AML and inform the efforts to improve anti-AML therapy. Finally, although the Review focuses on AML, we will also discuss the extent to which this disease can serve as a paradigm for understanding cancer evolution in general.

## The leukaemic stem cell

Peter Nowell was the first to describe cancer as an evolutionary process with parallels to Darwinian natural selection (Nowell, 1976). Complex organisms have evolved highly efficient systems to protect their cellular genomes from accumulating DNA mutations; however, such mechanisms are not impenetrable and cells slowly accumulate mutations over time, even in the absence of identifiable exogenous mutagens. The change from a normal to a cancer cell requires acquisition of multiple somatic mutations that collectively impart the malignant phenotype.

The potential for limitless self-renewal is one of the hallmarks of cancer (Hanahan and Weinberg, 2000), although it is recognised that this capacity is often restricted to a subpopulation of tumour cells, known as the cancer or leukaemia stem cells (CSC/LSC) (Lapidot et al., 1994). Individual cancer genomes are genetically heterogeneous and this is most likely to reflect heterogeneity at the level of LSCs. There is evidence that this is the case in acute lymphoblastic leukaemia (ALL). Transplantation of primary leukaemia cells into immunodeficient mice revealed variable competitive regeneration of subclones in patterns that reflect the diversity within the primary tumour (Anderson et al., 2011; Notta et al., 2011).

Normal HSCs, like other stem cells, are undifferentiated long-lived cells capable of asymmetric division, facilitating both self-renewal and the generation of differentiated progeny. In addition, HSCs can undergo either self-renewing (clonal expansion) or differentiating (clonal extinction) symmetric division (Pina and Enver, 2007). During normal haematopoiesis, the peripheral blood is estimated to have contributions from ~1000 HSCs (Catlin et al., 2011), whereas at any given time the majority of adult HSCs are in a quiescent state (Arai et al., 2004; Li and Clevers, 2010). On average, human HSCs are thought to divide once every 40 weeks (Catlin et al., 2011); however, blood cell production is a continuous process throughout life, with an adult human producing an estimated 10^11^ cells daily (Beerman et al., 2010). These properties make HSCs, like other tissue stem cells, prime targets for malignant transformation. Nevertheless, the fact that some mutations can transform differentiating cells suggests that HSCs might not be the only source of LSCs (Cozzio et al., 2003; Huntly et al., 2004).

## The mutational burden of cancer: drivers and passengers

**Fig. 1.**
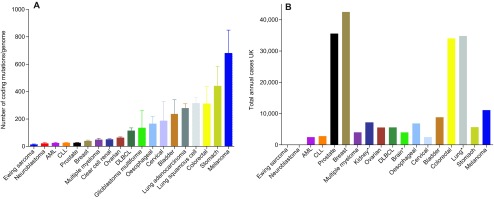
**Mutation burden and cancer incidence.** (A) Comparison of the mean number of non-coding mutations per genome in tumours of different tissues (raw data from Lawrence et al., 2013). Error bars show the standard error of the mean. (B) UK annual incidence of various malignancies [Cancer Registry Statistics, 2011; (www.ons.gov.uk) and the Cancer Research UK website (www.cancerresearchuk.org/cancer-info/cancerstats/)]. An asterisk (*) signifies that the incidence data refer to the tissue of origin, rather than the specific cancer subtype shown in A (e.g. lung cancer rather than lung squamous cell or adenocarcinoma). CLL, chronic lymphocytic leukaemia; DLBCL, diffuse large B-cell lymphoma.

The mutations present in a cancer cell genome accumulate throughout life and are the result of cell-intrinsic mutational processes and exposure to external mutagens. As a result, the median numbers of somatic mutations differ by more than 1000-fold between different cancer types (Alexandrov et al., 2013; Lawrence et al., 2013). It is estimated that about half of the variation in mutation frequencies can be explained by the intrinsic differences in somatic mutation rates between tissues (Lawrence et al., 2013); however, the number of somatic mutations can also vary by over 1000-fold between cancers of the same subtype (Alexandrov et al., 2013; Lawrence et al., 2013). AML has one of the lowest number of mutations per case of any adult cancer studied to date (Fig. 1), although the range varies widely between individual cases (Lawrence et al., 2013; The Cancer Genome Atlas Research Network, 2013).

A mutation that gives a cell a fitness advantage is termed a *driver* and one that has no effect on its fitness and/or growth characteristics is called a *passenger*. However, this binary classification of cancer mutations into drivers and passengers is context dependent. Tumour subclones compete with each other and with normal cells for ‘real estate’ and resources within the tissue microenvironment. Changes imposed on this ecosystem will alter the relative competitiveness of cancer cell clones. For example, after anti-cancer therapy, minor subclones able to survive treatment can regenerate the malignancy (Anderson et al., 2011; Ding et al., 2012). By contrast, mutations that confer drug resistance might be disadvantageous in the absence of treatment (Skaggs et al., 2006).

Mutations that in isolation have a neutral or even negative effect on long-term clonogenicity (passengers) might be ‘selected’ if they co-occur with a fitness-conferring mutation or are advantageous in the context of other mutations (epistatic effect). The persistence of passenger lesions in tumour cells is akin to genetic draft or the ‘hitchhiking’ effect seen in population genetics. These lesions are only detected in the final tumour because they happened to be present in a cell at the time of acquisition of the first or subsequent driver mutations. Factors that affect the number of passenger mutations include: (i) the number of cellular divisions between the zygote and the sequenced cancer cell; (ii) differences in susceptibility to somatic mutation; (iii) fidelity of DNA repair mechanisms; and (iv) differential exposure to mutagens. The highly variable number of passenger lesions both between and within subtypes of cancer affects the dynamics of clonal evolution (Nik-Zainal et al., 2012b; Welch et al., 2012).

It is worth noting that the driver versus passenger status remains formally untested for most cancer-associated mutations. For the time being, their recurrence rate within and between cancer types serves as a proxy for this status; that is, genes mutated in cancer more often than expected by chance are considered to be drivers. This is very likely to be an oversimplification, as it is difficult to determine what constitutes ‘chance’. For example, some very large genes are recurrently mutated by virtue of their size and others by virtue of their chromatin organisation (Lawrence et al., 2013).

## Mutational processes and rates

The biological processes that generate cancer-causing somatic mutations are being elucidated. A recent study characterised the somatic mutations in thousands of tumours from 30 cancer types and classified these according to the type of nucleotide change and its surrounding sequence. This revealed 21 distinct mutational ‘signatures’, only some of which related to known mutagens or intrinsic defects in DNA maintenance (Alexandrov et al., 2013). Some of these mutational signatures are shared across tumours of different types, whereas others are tumour specific. Signatures 1A and 1B were common to most cancer subtypes, and are the only signatures that have been identified in AML (Alexandrov et al., 2013). These signatures, thought to arise through spontaneous deamination of 5-methyl-cytosine, resulting in C>T transitions, were the only signatures with a strong positive correlation to patient age, suggesting that they accrued during life at a steady rate that is similar between individuals (Alexandrov et al., 2013). Spontaneous deamination of methylated CpG dinucleotides has previously been shown to accumulate at a relatively constant rate over time in primates (Kim et al., 2006) and explains the excess of A/T relative to C/G in genomes. Other signatures accumulate at varying rates between individuals and do not correlate with age, suggesting that they reflect differential exposures or susceptibilities to mutagens.

Many mutations in a cancer cell genome arise during DNA replication and cell division because of the minor intrinsic infidelity of the DNA replication and repair machineries. The rate of somatic mutation in normal human cells is difficult to measure, but is estimated to be in the order of 0.06–1.47×10^−9^ per genomic base pair per cell division (Lynch, 2010). The mutation rate varies between different regions of the genome; in data from whole genome sequencing of tumour-normal pairs, this difference was in excess of fivefold (Lawrence et al., 2013). Chromatin organisation (Schuster-Böckler and Lehner, 2012), replication timing (Chen et al., 2010; Woo and Li, 2012), strand (transcribed versus untranscribed) (Pleasance et al., 2010) and gene expression levels (Lawrence et al., 2013) correlate with regional mutation rates. Strikingly, the number of mutations in individual HSCs increases near linearly with age and is very similar to that found in *de novo* AML, suggesting that AML develops stochastically in a cell, which fortuitously accrues a transforming combination of mutations (Welch et al., 2012).

Mutations within a cell can influence the rate of acquisition of further lesions. After the initiating mutation, there might be a gradual accumulation of additional genetic alterations or accelerated progression due to genomic instability or catastrophic genetic events, including chromothripsis (the phenomenon by which hundreds to thousands of chromosomal rearrangements occur in a single event in localised and confined genomic regions in one or a few chromosomes) (Stephens et al., 2011) and kataegis (localized hypermutation of regions of the genome identified in some cancers) (Nik-Zainal et al., 2012a). Both of these processes are rare in AML (Alexandrov et al., 2013; The Cancer Genome Atlas Research Network, 2013). Copy number changes are uncommon in favourable and intermediate prognostic groups, even on high-resolution single-nucleotide polymorphism (SNP) array analysis (The Cancer Genome Atlas Research Network, 2013). However, copy neutral loss of heterozygosity (LOH) occurs relatively frequently, affecting mutations such as internal tandem duplication (ITD) of the gene *FLT3*, which leads to constitutive activation of the encoded receptor tyrosine kinase (Whitman et al., 2001). In fact, LOH for the *Flt3-ITD* mutation was a very early and almost universal event during leukaemia development in *Npm1c*/*Flt3-ITD* double heterozygous mice (Mupo et al., 2013), suggesting that this type of mutation can be rapidly acquired and selected for.

## Numbers of drivers and types of cancer

The total number of driver mutations that cooperate to induce a malignant phenotype is not well established and appears to differ among tumours. It is estimated that, in common adult epithelial tumours, there are on average 5–7 driver mutations; however, in haematopoietic malignancies this number might be lower (Stratton et al., 2009). This difference is likely to be at least partially attributable to the pattern and intensity of the mutational processes underlying each cancer type, rather than representing an intrinsic cellular characteristic. For example, a cancer arising through rare ‘background’ stochastic mutations might be more likely to arise through a small number of powerful mutations, whereas a cancer in which mutagenesis is avid might evolve through a larger number of weak mutations. This model predicts that the former type would be rarer, which is indeed broadly supported by observations on the total number of mutations in different cancer types (Fig. 1).

**Fig. 2.**
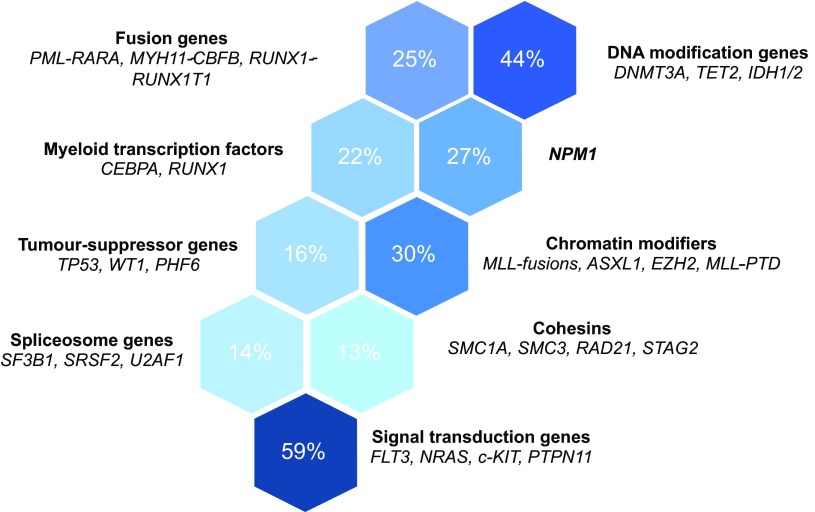
**Recurrent mutation groups in *de novo* AML.** Genes recurrently mutated in AML belong to distinct functional groups or pathways. The most prominent functional groups and genes associated with these are listed. The proportion of AMLs with mutations affecting each of these groups is displayed (data obtained from The Cancer Genome Atlas Research Network, 2013).

In AML, there is a relatively well-defined set of recurrent mutations, most of which fall into functional categories (Fig. 2) (The Cancer Genome Atlas Research Network, 2013). Whole genome or exome sequencing of 200 AMLs showed that nearly all had at least one and most had several recurrent mutations (The Cancer Genome Atlas Research Network, 2013). The variation in the identity of driver mutations is in keeping with the stochastic nature of myeloid leukaemogenesis, yet the identifiable patterns of co-occurrence and mutual exclusivity between specific mutations hint respectively at molecular synergy and redundancy between them (The Cancer Genome Atlas Research Network, 2013).

## How many and what types of mutations drive AML?

Gilliland and Griffin proposed the two-hit model of leukaemogenesis (Gilliland and Griffin, 2002). In this model, two lesions, each belonging to a different class, collaborate to cause AML when neither is sufficient to do so in isolation. Class I mutations such as *FLT3-ITD* or *N-RAS* mutations confer a proliferative advantage, but have no effect on differentiation. Class II mutations (represented by specific fusion genes in the original model) impair haematopoietic differentiation and subsequent apoptosis. The initiating lesions in these AMLs are thought to be class II mutations, for example *PML/RARα* and *MLL* gene fusions, whereas class I mutations are typically later events. This model has provided a useful framework to conceptualise the pathogenesis of AML as a disease in which differentiation is blocked and proliferation is increased. Although most of the recently identified mutations (Fig. 2) do not fit neatly into one of the two classes, they are thought to synergistically produce the equivalent effects.

The number of identifiable driver mutations differs between AML cases. In the whole genome/exome study of 200 AMLs conducted by The Cancer Genome Atlas (TCGA) Research Network, the authors describe a mean of 13 (range 0–51) tier 1 (coding, splice site and RNA gene) mutations (The Cancer Genome Atlas Research Network, 2013). On average, five of these were in genes that are recurrently mutated in AML. The number of recurrent tier 1 mutations was lower in the presence of specific translocations, whereas higher numbers were observed in cases with *RUNX1–RUNX1T1* fusions and those without fusion genes (The Cancer Genome Atlas Research Network, 2013). Co-occurrence analysis showed that some common mutations in genes including *DNMT3A*, *CEBPA*, *IDH1/2*, *NPM1* and *RUNX1*, which have more or less well-defined epigenomic consequences, were mutually exclusive of transcription factor fusions. The authors proposed that these mutations might have a role in the initiation of AML (The Cancer Genome Atlas Research Network, 2013).

Another important set of AML mutations are those involving large chromosomal gains or losses. The commonest amongst these are deletion 5q, monosomy 7, and trisomies of chromosomes 8, 11 and 13. There is strong evidence that changes in the expression of deleted or amplified genes located in these large regions drive leukaemogenesis (Shlush et al., 2014) and influence patient prognosis (Tutt et al., 2010). Additionally, array-based genomic studies of AML have identified a number of smaller genomic regions of copy number aberration, even in karyotypically normal AML (Casas et al., 2004; Itzhar et al., 2011). Some of these lesions, including trisomy 8 and small deletions affecting TET2 (the gene for a DNA methylcytosine dioxygenase) and DNMT3A (which encodes DNA methyltransferase A), can be seen in the blood of haematologically normal individuals, suggesting that they represent early events in leukaemogenesis (Jacobs et al., 2012; Laurie et al., 2012).

Although difficult to validate given the vastness of mammalian genomes, evidence from mouse models suggests that as few as two highly complementary mutations can be sufficient to generate AML (Mupo et al., 2013; Wartman et al., 2011). In a knock-in mouse model, the combination of *Npm1c* and *Flt3-ITD* caused universal leukaemia, with all mice becoming moribund in 31–68 days (Mupo et al., 2013). In another model, the co-expression of *PML-RARα* and *Jak1 V657F* mutations in mice resulted in rapid onset of acute promyelocytic leukaemia (APL)-like leukaemia, with a mean latency of 35 days (range 28–52 days) (Wartman et al., 2011). Compared to single-mutant controls, both models demonstrated a highly increased penetrance and a markedly accelerated disease onset in double mutant mice. Although these observations suggest that specific combinations of two mutations might be sufficient to drive AML, the possibility that additional mutations are rapidly acquired cannot be ruled out. In fact, in the former model most AMLs displayed acquired LOH for *Flt3-ITD*.

Similarly, human sequencing data describe many AMLs with only one or two identifiable driver mutations. The difficulty of interpreting this is compounded by the real possibility that driver mutations were missed or misclassified as passengers because of their rarity. Nevertheless, it remains possible that specific combinations of two mutations might be sufficient for leukaemogenesis, although most cases harbour three or more identifiable drivers at the time of clinical presentation (Welch et al., 2012). Whole genome sequencing of 12 human samples of APL included one case in which *FLT3-ITD* and *PML-RARα* were the only recurrent cancer-associated tier 1 somatic mutations in the tumour genome (Welch et al., 2012). The possibility that additional non-recurrent driver mutations contributed to pathogenesis cannot be excluded; in a further four cases of APL with these two mutations, additional cancer-associated tier 1 mutations were identified. However, in a mouse model, *PML-RARα* and *FLT3-ITD* induced an APL-like disease with complete penetrance and a short latency (Kelly et al., 2002). These apparent discrepancies hint at some as yet poorly understood factors driving individual leukaemias, such as undiscovered non-coding somatic mutations, cell extrinsic factors and heritable susceptibilities (discussed later).

## The timeframe of AML evolution

Available evidence suggests that cancer evolution is an unpredictable process with a highly variable rate of progression (Stratton et al., 2009). AML is an uncommon cancer whose incidence rises with age, but can occur at any age, with 15% of cases in people under 40 (AIHW, 2013; Bhayat et al., 2009; Dores et al., 2012; Shah et al., 2013). The rarity of the disease mirrors the small mutational burden of AMLs and might reflect a paucity of external mutagens in the HSC niche or an unusual level of protection against mutation. One explanation for the latter is the ability of a small fraction of HSCs to sustain haematopoiesis at any time, allowing HSCs to remain quiescent for most of their lifespan and, in so doing, reducing their total number of divisions. This is only possible because of the very high proliferative capacity of later progenitors, whose limited lifespan and self-renewal minimises their own risk of transformation.

Pre-leukaemic clones arise with surprising frequency during foetal development. The *in utero* acquisition of leukaemogenic mutations was first reported in concordant twins with ALL, whose haematopoietic cells shared a unique somatic rearrangement involving the *MLL* gene (Ford et al., 1993). Subsequently, clonotypic *RUNX1–RUNX1T1* (*AML1–ETO*) fusion sequences were detected in Guthrie spots in cases of childhood AML (Wiemels et al., 2002). However, the prevalence of detectable *RUNX1–RUNX1T1* and *TEL–RUNX1* in cord blood is 100-fold greater than the risk of the corresponding leukaemia, and the frequency of positive cells (10^−4^ to 10^−3^) indicates substantial clonal expansion of the abnormal progenitor population (Mori et al., 2002). This is because these fusion genes are not sufficient for disease development, as indicated by protracted post-natal latencies, non-concordant phenotypes in monozygotic twins (Wiemels et al., 1999; Wiemels et al., 2002) and the lack of overt leukaemia in transgenic mice (Rhoades et al., 2000). Therefore, secondary genetic events appear necessary for tumour development. It is unknown whether foetal acquisition of *RUNX1–RUNX1T1* can lead to adult-onset AML, but it is possible that long-lived HSCs progress only in later life, for example following chemotherapy in therapy-related AML. In fact, adults treated for *RUNX1–RUNX1T1*-positive AML can exhibit persistence of the fusion in the blood for years in the absence of disease relapse (Kusec et al., 1994; Miyamoto et al., 1996).

The presence of detectable oncogenic mutations in blood in the absence of haematological disease is not unique to childhood. For example, inactivating somatic mutations affecting *TET2* were identified in 10 of 182 females aged over 65 with skewed X-chromosome inactivation patterns (XCIP) and normal haematopoietic parameters (Busque et al., 2012). Mice with *Tet2* deletion exhibit increased HSC self-renewal potential, without detectable changes in standard haematological parameters, paralleling what happens in humans (Moran-Crusio et al., 2011; Quivoron et al., 2011). After follow up of seven *TET2* mutant individuals for at least 5 years, one developed evidence of a haematological malignancy: a *JAK2V617F* mutant myeloproliferative neoplasm (MPN) (Busque et al., 2012).

The above findings show that somatic mutations, a universal feature of normal ageing, can drive the expansion of individual HSCs to the point of dominating haematopoiesis without causing disease. Nevertheless, the onward development of a haematological malignancy, although not inevitable, becomes much more likely. This behaviour is not unique to *TET2* mutations, but is also a feature of other somatic mutations, such as large chromosomal deletions or amplifications, which also increase in frequency with age (Jacobs et al., 2012; Laurie et al., 2012; Schick et al., 2013). In fact, there is a 5–10-fold increase in the risk of developing a haematological malignancy in the decade after the detection of mosaicism for such chromosomal changes in blood leukocyte DNA (Laurie et al., 2012; Schick et al., 2013).

Some studies that have analysed the clonal composition of blood from healthy women using X inactivation markers suggest that this is stable over time even in the elderly (Prchal et al., 1996; Swierczek et al., 2008). However, a study of the serial composition of copy number variants (CNVs) in individuals without diagnosed haematopoietic disorders showed clear fluctuations in the proportion of nucleated blood cells with aberrations over time (Forsberg et al., 2012). In one person with a 20q deletion, the variant was barely detectable at 71 years of age, accounted for 50% of cells at 75 years, but only 36% at 88 years of age (Forsberg et al., 2012). In a longitudinal study of colony-stimulating factor 3 receptor gene (*CSF3R*) mutations in congenital neutropenia, the independent acquisition of several different *CSF3R* mutations in different cells was demonstrated (Beekman et al., 2012; Campbell et al., 2010). Serial analysis of patient samples showed that one mutation or clone dominates at a time, but new mutations are able to replace previously dominant ones and mutations that fall below the limit of detection are sometimes detectable in subsequent samples (Campbell et al., 2010). It is unknown whether the clonal expansion of cells containing genetic abnormalities is always due to positive selection or reflects stochastic fluctuations in the numbers of HSC progeny or cycles of quiescence and active contribution to haemopoiesis by different HSCs.

## Role of the germline genome

There is good evidence that an individual’s constitutional genome has an impact on both mutation rate and the fate of mutant cells. Individuals with familial myelodysplastic syndromes (MDS), which is associated with mutations in the *RUNX1* gene, have a median AML incidence of 35% in carriers, but this varies greatly between families, as does the age of onset, which ranges from childhood to old age even within the same family (Owen et al., 2008). As the *RUNX1* mutations are shared by family members, the variable penetrance and age of onset of haematopoietic malignancy indicate that either the rate of acquisition of cooperating mutations and/or their impact are variable between individuals.

There is ample evidence from mouse models of the interaction between the constitutional genome and cancer phenotype in both solid (Diwan et al., 1986) and haematopoietic (Potter and Wiener, 1992; Yamada et al., 1994) malignancies. One example is the strain-specific effect of insertional mutagens such as the Graffi murine leukaemia virus (MuLV). When mice were inoculated with similar doses of two closely related Graffi MuLV strains, the latency to tumour development differed significantly between BALB/c, NFS and FVB/N mice (Voisin et al., 2006). Furthermore, the same viral strain produced a different tumour spectrum in the three mouse backgrounds (Voisin et al., 2006).

In human disease, there is also evidence that the constitutional genome affects the risk of acquiring specific somatic mutations. A well-documented example is the association of the 46/1 (or GGCC) *JAK2* haplotype with *JAK2V617F* mutant MPN (Jones et al., 2009; Olcaydu et al., 2009). The finding that the *JAK2V617F* mutation preferentially appears in a particular haplotype of *JAK2* has been validated across European (Jones et al., 2009; Olcaydu et al., 2009), Chinese (Wang et al., 2013) and Japanese (Tanaka et al., 2013) populations. The 46/1 haplotype is common, with a frequency of 24% in European populations and an odds ratio of developing MPN of 3–4 (Jones et al., 2009). The basis of this association remains unknown, but might be due to a possible association of the 46/1 haplotype with higher *JAK2* expression in a key cell type.

## Linear versus branching evolution and clonal hierarchy

**Fig. 3.**
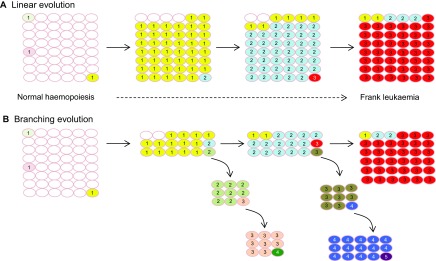
**Linear and branching clonal evolution.** (A) Linear evolution. Sequential dominant clones (clonal sweep) result in a linear architecture with stepwise accumulation of driver mutations. The final tumour carries all mutations arising during the evolutionary history and overwhelms earlier clones carrying only some of the mutations. (B) Branching evolution. The final leukaemia might be dominated by a single clone, but clones arising through divergent mutational pathways are also evident. Small subclones might fall below the limit of detection, in which case the complexity of branching is underestimated. Smaller fitness effects of mutations and faster acquisition favour branching versus linear evolution. Numerals indicate the number of mutations in cells. Cells carrying identical mutations are represented in the same colour.

Cancer dynamics depend on the rate of acquisition of fitness-conferring mutations, the relative selective advantage they give and the size of the susceptible cell population. A mutation that confers a strong selective advantage could allow a clone to expand and dominate the haematopoietic compartment in a ‘selective sweep’, especially if there is a long lag time before additional driver mutations occur. With sequential dominant clones, leukaemia evolution would be represented by an essentially linear architecture with stepwise accumulation of driver mutations (Fig. 3A). However, deep sequencing methods have revealed that cancers, including AML, are characterised by significant mutational complexity and that the diversity and relative dominance of subclones varies throughout the course of disease (Anderson et al., 2011; Campbell et al., 2008; Campbell et al., 2010; Ding et al., 2012; Gerlinger et al., 2012; Nik-Zainal et al., 2012b; Notta et al., 2011). The subclones with the highest numbers of genetic abnormalities are not necessarily numerically dominant within the tumour (Anderson et al., 2011; Jan et al., 2012; Walter et al., 2012). Cancers can be traced back to a single cell, but the continuous acquisition of mutations and associated expansions in population sizes dramatically increase genetic and clonal heterogeneity, and it is likely that most cancers evolve with a complex branching architecture (Fig. 3B).

In deep sequencing studies of mixed tumour cell populations, the variant allele frequencies can be used to estimate the size of subclones. Whole genome sequencing of 24 primary AML samples revealed between one and four clusters of mutations based on variant allele frequency, although the number of variants specific to individual subclones was small (average of only 40) (Welch et al., 2012). Most AML-associated mutations are generally shared by all leukaemic clones, as the initiating lesion arises in a cell with a mutational history (Welch et al., 2012). Therefore, it does not seem surprising that subclone-specific single nucleotide variants (SNVs) accounted for only 14% of the total (Welch et al., 2012). By contrast, in tumours with higher mutational burdens, the proportion of SNVs specific to subclones can be much higher (Gerlinger et al., 2012; Nik-Zainal et al., 2012b).

Although the prevailing dogma is that the evolution of cancer occurs through a complex branching pattern of mutation acquisition (Greaves and Maley, 2012), there is evidence for both linear and branching pathways prevailing in individual AMLs. A comparison of paired primary and relapsed AML samples revealed two patterns of clonal evolution during relapse (Ding et al., 2012). In some cases, only a single mutation cluster was found in the primary tumour. In these cases, the single clone gained additional mutations at relapse, consistent with a linear pattern of evolution, although minor branching subclones could have been missed. In the remaining cases, multiple mutation clusters corresponding to different subclones were detected in the primary sample. A subclone survived therapy, gained additional mutations and expanded at relapse (branching evolution). In comparison to primary tumour mutations, there was an increase in transversions among relapse-specific mutations, thought to arise from DNA damage caused by cytotoxic therapy (Ding et al., 2012).

Similarly, two studies comparing acquired copy number aberrations (CNAs) and copy neutral LOH in paired diagnosis and relapse samples in *NPM1* mutant AML (Krönke et al., 2013) and unselected cases of AML (Parkin et al., 2013) found that reemergence or evolution of a founder or ancestral clone is typical in relapsed AML (Krönke et al., 2013). This is in contrast to findings in ALL, in which genetically distinct clones are occasionally observed (Mullighan et al., 2008).

These studies focus on clonal heterogeneity at the genetic level, but there is good evidence that non-genetic mechanisms contribute to the functional heterogeneity of cancer cells. For example, the repopulation kinetics of single-cell-derived clones that shared a common genetic lineage were highly variable in a murine xenotransplant model of colorectal cancer (Kreso et al., 2013). Mechanisms probably include epigenetic and environmental effects, such as differential exposure of CSCs to therapeutic agents and growth factors.

## Genotype-phenotype correlations and myeloid malignancies

**Fig. 4.**
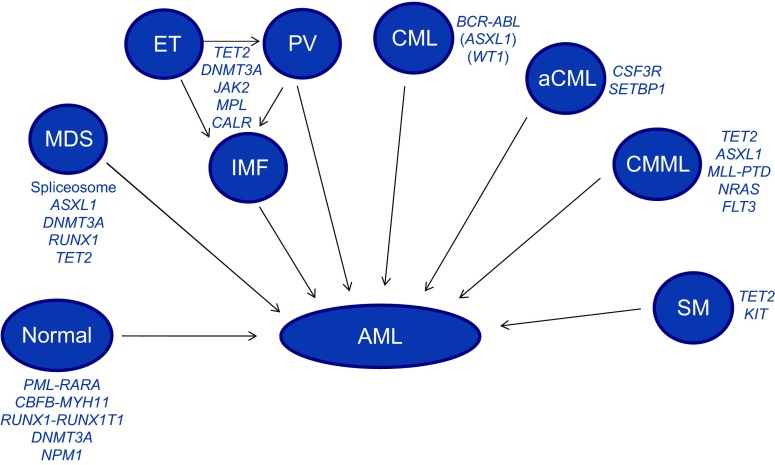
**Common mutations in *de novo* and secondary AML.** A number of clonal blood disorders with a myeloid phenotype are represented. Each of these disorders is characterised by recurrent mutations in specific genes, some of which are shared between several different phenotypes (e.g. *TET2*). All of these disorders can transform to secondary AML upon acquisition of additional somatic mutations. When AML arises in the absence of an antecedent clonal blood disorder, it is known as primary AML. aCML, atypical CML; CML, chronic myeloid leukaemia; ET, essential thrombocythaemia; IMF, idiopathic myelofibrosis; PV, polycythaemia vera; SM, systemic mastocytosis.

Many common mutations driving myeloid neoplasms are found in several phenotypically distinct diseases (Fig. 4). For example, *TET2* mutations are found recurrently in AML, MDS, MPN and chronic myelomonocytic leukaemia (CMML), as well as occurring in lymphoid tumours (Delhommeau et al., 2009; Quivoron et al., 2011). This raises two important questions: first, to what extent can the disease phenotype be deduced from its complement of somatic mutations and, second, how do shared initiating mutations evolve into distinct neoplasms?

Although the LSC is the cell of origin for AML, selective pressures are applied to tumour cells at all stages of differentiation in the mixed tumour population. Itzykson et al. analysed candidate genes in single-cell-derived colonies from CMML patients to characterise the distribution of mutations at various stages of progenitor differentiation (Itzykson et al., 2013). Subclones with a greater number of mutations were over-represented in the granulocyte-monocyte progenitors (GMP) compared to the HSC/multipotent progenitor (MPP) compartment, even though CMML is a disease of HSC origin and clonal dominance of the malignant clone is evident at the HSC/MPP stage (Itzykson et al., 2013). Therefore, it seems that these mutations, which are present in only some of the LSCs, impart an additional clonal advantage to differentiating progeny. A comparison of *TET2* mutant CMML and MDS samples found that the peripheral monocyte count correlated with the proportion of *TET2* mutated CD34^+^/CD38^−^ cells, suggesting that the extent of dominance of the *TET2* mutated clone in the HSC/MPP compartment influences the clinical phenotype (Itzykson et al., 2013). However, the serial analysis of samples from individual patients also provided evidence that changes in the clonal composition of the HSC/MPP compartment are not always reflected in the disease phenotype. For example, some patients showed a significant increase in the proportion of double-mutant HSC/MPP clones over time, even though the clinical phenotype was unchanged (Itzykson et al., 2013).

Together, such findings indicate that varied selective pressures and fitness determinants drive clonal outgrowth at different stages of the myeloid stem and progenitor cell hierarchy. This is relevant to sequencing studies, as the distribution of mutations detected in the mass tumour population will not necessarily reflect their frequency in LSCs. Furthermore, when evaluating treatment it is important to recognise that therapies that remove the proliferative advantage of a subclone during differentiation can have a short-term phenotypic benefit by reducing tumour bulk, but will not necessarily have the same impact on LSCs.

## Initiating mutations and order of acquisition

The few human studies that track mutations in sequential AML samples compare relapsed versus primary tumours, or secondary AML versus a preceding haematological disorder, rather than profiling the pre-leukaemic evolution of primary or *de novo* AML (Ding et al., 2012; The Cancer Genome Atlas Research Network, 2013; Walter et al., 2012). The initiating lesion is definitively known only in familial AML; however, the dynamics of clonal evolution are likely to be different in this situation, in which all haemopoietic stem and progenitor cells (HSPCs) carry the initiating mutation, compared with sporadic AML. Our understanding of initiating mutations in *de novo* AML is derived from studies of mutational allelic burden, stability of mutations through the disease course, patterns of co-occurrence between mutations, specificity for a particular AML phenotype and mechanistic studies of the properties of specific mutations. Generally, it is thought that proliferative (type I) mutations are later events that cooperate with a variety of initiating lesions to produce disease. However, it is clear that at least some lesions can occur as either early or late events in the same tumour type, suggesting they are not acquired in any strict order (Anderson et al., 2011). In AML, there are examples of ‘early’ mutations lost at relapse and ‘late’ mutations that are acquired first.

Mutations in *NPM1*, the gene encoding the nucleolar phosphoprotein nucleophosmin, have been considered early events in *de novo* AML, largely because of their stability through the disease course and their mutual exclusivity with chromosomal translocations, the best-established type of initiating mutation. However, in a study comparing CNAs and recurrent mutations in paired diagnosis and relapse samples of 53 *NPM1* mutant AMLs, mutations in *DNMT3A* were the most stable lesion. Persistence of *DNMT3A* was found in five patients who lost the *NPM1* mutation at relapse, suggesting that *DNMT3A* preceded *NPM1* mutations (Krönke et al., 2013). However, there was also a single case in which *DNMT3A* was lost at relapse and the *NPM1* mutation was maintained, which implies that the mutation order is not strict. More recently, mutations in *DNMT3A*, but not *NPM1*, were identified in pre-leukaemic HSCs from patients with double DNMT3A/NPM1 mutant AML, further supporting the leukaemia-initiating pedigree of mutant DNMT3A (Shlush et al., 2014).

The order of mutation acquisition can also be determined by comparing the patterns of co-occurring mutations in residual HSCs or leukaemia cells (Itzykson et al., 2013; Jan et al., 2012). In one study, residual HSCs were screened for patient-specific mutations identified by tumour exome sequencing in six patients with *de novo*, *FLT3-ITD* mutant, normal karyotype AML (Jan et al., 2012). Many AML-associated mutations, including *NPM1*, *TET2* and *SMC1A*, were detectable in the residual HSC, but others, such as *FLT3-ITD* and *IDH1*, were not, indicating that these were probably late events. The population of residual HSCs showed varying allele frequencies for each of the detectable mutations. By comparing the patterns of mutations at the single-cell level, researchers were able to reconstruct the phylogenetic tree in several cases (Jan et al., 2012).

So why are some mutations more often early and others more often late events? It is very likely that, in the great majority of AMLs, the initiating mutation happens stochastically. However, this might alter the probability and type of secondary mutations en route to a malignancy. Potential mechanisms include a restriction in the cellular pathways through which secondary mutations could imbue additional fitness, but are not limited to this. For example, induced changes in the epigenetic programme or the microenvironment might alter the phenotypic consequences of secondary mutations or the nature of selective pressures. Evidence of convergent evolution in multiple tumour types (Anderson et al., 2011; Gerlinger et al., 2012) suggests that (i) those mutations are targeted by a specific mechanism of mutation, for example, the off-target effects of activation-induced deaminase (AID), or (ii) such mutations are recurrently selected because of their strong fitness advantage in a situation of high mutational diversity (parallel evolution) or (iii) the spectrum of cooperating lesions is severely limited in the context of pre-existing mutations.

**Fig. 5.**
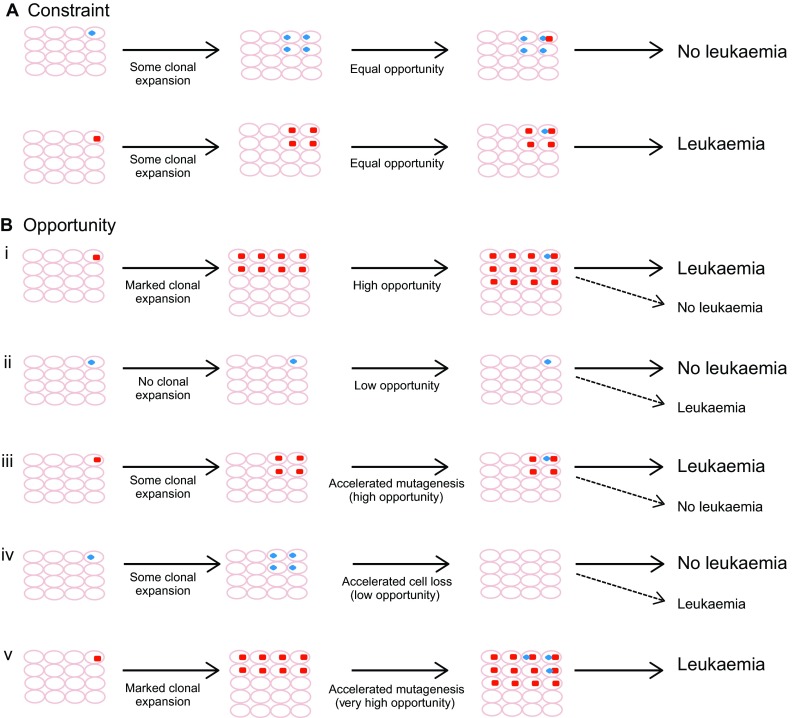
**Order of acquisition: constraint or opportunity?** In many cancers, including AML, specific driver mutations are usually acquired early in the process of clonal evolution, whereas others are acquired late. Here we use the simplified example of two mutations that represent the only two driver events in AML. During the evolution of AML, mutation X (red square) is recurrently acquired first and mutation Y (blue oval) second. This pattern could be due to a strict requirement for a specific order in which mutations are acquired (constraint, panel A) or could simply reflect the statistical likelihood that mutations are acquired in this order (opportunity, panel B). We speculate that the key variables behind the concept of ‘opportunity’ are the number and longevity of cells susceptible to transformation (clonal size, represented here by the number of cells that inherit the mutation) and the speed with which additional mutations are acquired (mutagenesis rate). In panel Bi, mutation X leads to a marked clonal expansion in the progeny of the leukaemia-initiating cell (LIC). In turn, this increases the likelihood of a cell subsequently acquiring mutation Y and the development of leukaemia (solid arrow). Nevertheless, the development of leukaemia is not inevitable (dashed arrow). In panel Bii, mutation Y is acquired first in the putative LIC and does not facilitate the generation of progeny susceptible to transformation, such that the subsequent acquisition of mutation X is unlikely (solid arrow), but not impossible (dashed arrow). In panel Biii, mutation X has a neutral effect on the generation of LIC progeny, but causes accelerated mutagenesis and thus makes the likelihood of subsequent acquisition of mutation Y higher. In panel Biv, mutation Y is acquired first and here it has a neutral effect on initial LIC clonal size, but does lead to subsequent cell loss (e.g. by accelerating senescence), therefore markedly reducing the opportunity for acquiring additional mutations. Again, this eventuality, although unlikely, is not impossible. Finally, in panel Bv, mutation X leads to both clonal expansion and accelerated mutagenesis, making the development of leukaemia very likely or even inevitable. By the same token, a mutation with the opposite effects (i.e. no LIC clonal expansion or enhanced cell loss, and low mutagenesis rate) would make leukaemia very unlikely or impossible.

It is probable that there are no set rules or ‘constraint’ governing the order of acquisition of mutations in AML, but rather that the specific consequences of individual mutations make them more or less likely to facilitate subsequent evolution to leukaemia. In other words, the bias described in the order of acquisition might reflect ‘opportunity’, rather than being an absolute requirement (Fig. 5). Consider an example in which mutations ‘X’ and ‘Y’ cause AML when they co-occur within the same HSC, but have only modest effects when they occur in isolation. Mutation ‘X’ augments clonal expansion of the HSC, that is, the leukaemia-initiating cell (LIC), whereas mutation ‘Y’ does not. When mutation ‘Y’ occurs first, the number of single-mutant HSCs susceptible to a second transforming hit is very small, thus making transformation unlikely. In the absence of an unlikely second lesion occurring in the few mutant HSCs, they persist in limited number or become quiescent, or even senesce with time. By contrast, when mutation ‘X’ occurs first, the pool of single-mutant HSCs is expanded and this ensures that at least some of the progeny remain in cycle thereafter. The likelihood of a mutated HSC acquiring a second hit is now much higher.

This illustrative example does not take into account many other variables that can operate to affect the order of mutation acquisition, but serves to outline the concept of ‘opportunity’. For instance, a mutation that dramatically increases the rate of acquisition of further mutations would be predicted to increase the likelihood of developing AML; however, this might not be the case if the same mutation also leads to rapid senescence of the host cells. Therefore, ‘opportunity’ in this context is a function of (i) the mutation rate, (ii) the number of cells susceptible to transformation and (iii) the time these cells remain susceptible. Such concepts can be difficult to establish from human samples. Mutations in *TET2*, *DNMT3A* and *NPM1* are thought to be early events in human AML (Jan et al., 2012; The Cancer Genome Atlas Research Network, 2013) and in mouse models mutant *Tet2*, *Dnmt3a* and *Npm1* cause increased stem cell self-renewal, akin to mutation ‘X’ (Challen et al., 2012; Moran-Crusio et al., 2011; Quivoron et al., 2011; Vassiliou et al., 2011). In fact, there is now good evidence that *DNMT3A* mutations do expand the pre-leukaemic HSC (LIC) clone during the evolution of human AML. In murine transposon insertional mutagenesis models, leukaemia becomes inevitable following the clonal expansion of cells with an initiating mutation in the setting of increased mutagenesis, whereas the clonal size and order of acquisition are both key variables in leukaemia evolution in these models (Vassiliou et al., 2011; C.S.G. and G.S.V., unpublished observations).

## Conclusions and implications for therapy

As is the case for many cancers, efforts to develop improved therapies for AML cannot ignore its molecular heterogeneity and subclonal structure. This brings into focus the choice between therapies targeting specific genetic mutations and those operating on shared targets, as well as the need to combine multiple therapies to treat diverse subclones. For example, it is yet to be determined whether the best approach will be to use a combination of targeted therapies at the earliest possible time, akin to combination antiretroviral therapy in HIV (Goldie and Coldman, 1984). There are theoretical advantages of attacking an identifiable (pre)leukaemic clone early, but the picture is complicated in AML as therapy might induce further genetic changes and drive disease evolution (Ding et al., 2012). Recent advancements in understanding the clonal evolution of leukaemia and other tumours have important implications for the development of novel therapeutic approaches, some of which are discussed below.

Passenger mutations can have disadvantageous effects on tumour cells and as they accumulate they can alter the course of neoplastic progression. Tumour progression depends on driver fitness outweighing any negative effects of passengers. Mathematical models predict that exacerbating the deleterious effects of passenger mutations or accelerating the mutation rate could actually be exploited in cancer treatment (McFarland et al., 2013). In keeping with this strategy, results from animal cancer models demonstrate that excessive chromosomal instability might have a tumour suppressive role (Weaver et al., 2007). In human disease, a synthetic lethality approach using poly ADP ribose polymerase (PARP) inhibitors in breast cancers with inherited defects in DNA repair is showing promise. The concern for such approaches is that tumour heterogeneity can also lead to faster tumour progression (Aktipis and Nesse, 2013) or even a surprising evolutionary viability of mutator phenotypes (Datta et al., 2013). It remains to be determined whether such an approach has a role in AML.

Another novel approach under investigation is that of adaptive therapy (Gatenby et al., 2009). As opposed to conventional therapeutic approaches aiming to induce lethal toxicity of tumour cells, in this model, therapy is continuously adjusted to achieve a fixed tumour population. This approach is founded on the theory that when resistant clones arise they are typically small in untreated tumours because of the ‘fitness cost’ of the resistant phenotype. Therapy designed to kill as many cells as possible promotes rapid outgrowth of resistant populations, by removing the inhibitory effect of competing tumour clones. In contrast, if chemo-sensitive cells are allowed to survive, they suppress the proliferation of resistant populations. Using mathematical and *in vivo* modelling of a solid tumour, Gatenby et al. found that progressively lower doses of chemotherapy and increased dose intervals were required to maintain the target tumour burden (Gatenby et al., 2009). Such adaptive approaches, although not curative, might prolong survival when curative therapy is unavailable or inappropriate. Furthermore, they predict that maximal tumour sensitivity to dose-intense drugs will occur after time, raising the possibility that delaying the attempt to cure until after a period of therapy that maintains a constant tumour size might be more effective (Gatenby et al., 2009).

The Darwinian model also emphasises the importance of the micro-environment on tumour growth dynamics. Both normal and cancer cells can provide growth signals or other fitness-enhancing factors for cancer cells. For example, in AML, leukaemia-derived M-CSF and IL-10 instruct stromal cells to secrete Gas6, which is the ligand for the TAM family tyrosine kinase receptor Axl (Ben-Batalla et al., 2013). In conjunction with autocrine or paracrine Gas6, Axl upregulation is thought to have a role in the chemoresistance of AML cells and this feedback loop provides a potential therapeutic target (Ben-Batalla et al., 2013). It is argued that the generation of cells other than cancer stem cells in the tumour population might have a positive influence on the fitness of tumour-propagating cells (Sprouffske et al., 2013). Thus, approaches that focus on cells other than the LSC warrant further investigation.

Overall, the evidence from AML shows that relapse occurs because of re-emergence or evolution of a founding or ancestral clone (Ding et al., 2012; Krönke et al., 2013; Parkin et al., 2013), identifying the genetic diversity of LSCs at diagnosis as a fundamental problem. Targeted therapy will be ineffective unless all clones with leukaemogenic potential are treated. Going forward, our increased knowledge of clonal dynamics and architecture can be harnessed to increase treatment success in AML and in other cancers. It is probable that other cancer types and even individual cancers have their own idiosyncrasies with regards to their evolution and clonal diversity; however, many of the principles outlined here are likely to apply and the body of knowledge amassed for AML can inform efforts to understand and most importantly to treat many other malignancies.
